# Analysis of Patents Issued in China for Antihyperglycemic Therapies for Type 2 Diabetes Mellitus

**DOI:** 10.3389/fphar.2019.00586

**Published:** 2019-05-21

**Authors:** Wei Zhu, Wen Huang, Zhiqiang Xu, Mengda Cao, Qiaoli Hu, Chen Pan, Miao Guo, Ji-Fu Wei, Hongyu Yuan

**Affiliations:** ^1^Research Division of Clinical Pharmacology, the First Affiliated Hospital of Nanjing Medical University, Nanjing, China; ^2^Department of GCP Office, Nanjing First Hospital, Nanjing Medical University, Nanjing, China; ^3^School of Basic Medicine and Clinical Pharmacy, China Pharmaceutical University, Nanjing, China

**Keywords:** type 2 diabetes mellitus, antihyperglycemic, patent, review, therapy

## Abstract

Type 2 diabetes mellitus (T2DM) is prevalent, with a dramatic increase in recent years. Moreover, its microvascular and macrovascular complications cause significant societal issues. The demand for new and effective antidiabetic therapies grows with each passing day and motivates organizations and individuals to pay more attention to such products. In this article, we focused on oral antihyperglycemic drugs patented in China and introduced them according to their antihyperglycemic mechanisms. By searching the website of State Intellectual Property Office of the People’s Republic of China (http://www.sipo.gov.cn), 2,500 antihyperglycemic patents for T2DM were identified and analyzed. These consisted of 4 patents for derivatives of herbal extracts (0.2%), 162 patents for herbal extracts (6.5%), 61 compositions for traditional Chinese medicine (TCM) (2.4%), 2,263 patents for synthetic compounds (90.5%), and 10 (0.4%) patents of the combination of synthetic compounds and TCM. As the most common drugs for diabetes mellitus, synthetic compounds can also be classified into several categories according to their working mechanisms, such as insulin secretion promotor agents, insulin sensitizer agents, α-glucosidase inhibitors, and so forth. This article discussed the chemical structure, potential antihyperglycemic mechanism of these antihyperglycemic drugs in patents in China.

**Expert opinion:** Insulin sensitivity and β-cell function could be improved by weight loss to prevent prediabetes into T2DM. However, 40–50% patients with impaired glucose tolerance (IGT) still progress to T2DM, even after successful long-term weight loss.

Antihyperglycemic remedies provide a treatment option to improve insulin sensitivity and maintain β-cell function. Combination therapy is the best treatment for diabetes. Combination therapy can reduce the dosage of each single drug option, and avoid the side effects. Drugs with different mechanisms are complementary, and are better adapted to patients with changing conditions. Classical combination therapies include combinations such as sulfonylureas plus biguanides or glucosidase inhibitors, biguanide plus glucosidase inhibitors or insulin sensitizers, insulin treatment plus biguanides or glucosidase inhibitors. The general principle of combination therapy is that two drugs with different mechanisms are selected jointly, and the combination of three types of hypoglycemic drugs is not recommended. After reading a large amount of literature, we have rarely found a case of three oral hypoglycemic agents, which may mean that the combination of three oral hypoglycemic agents is unnecessary and has unpredictable risks. There is no objection to the idea of multi-drug therapy. But multiple drugs can only be used when it shows a significant benefit to the patients. Combined use of multiple antidiabetic drugs poses a risk to patients due to drug interactions and overtreatment.

## Introduction

Diabetes mellitus is classified as type 1 diabetes (T1DM), type 2 diabetes mellitus (T2DM), gestational diabetes, and other types of diabetes mellitus in terms of their different etiology, genetics, and clinical manifestation (American Diabetes Association, 2014). In 2000, there were 150 million patients with diabetes mellitus, and the number increased to 200 million in 2010 (Mulnier et al., 2006). Among these, T2DM attracts more attention due to its high morbidity and mortality. It accounts for 90% of diabetes mellitus patients (Rao Kondapally Seshasai et al., 2011; Rahelić, 2016; Zheng et al., 2018; Deng et al., 2016). In China, the overall prevalence of T2DM was 7.9% (Han et al., 2017). T2DM is diagnosed by clinical manifestations of diabetes mellitus and elevated plasma glucose in laboratory tests.

**Figure 1:**
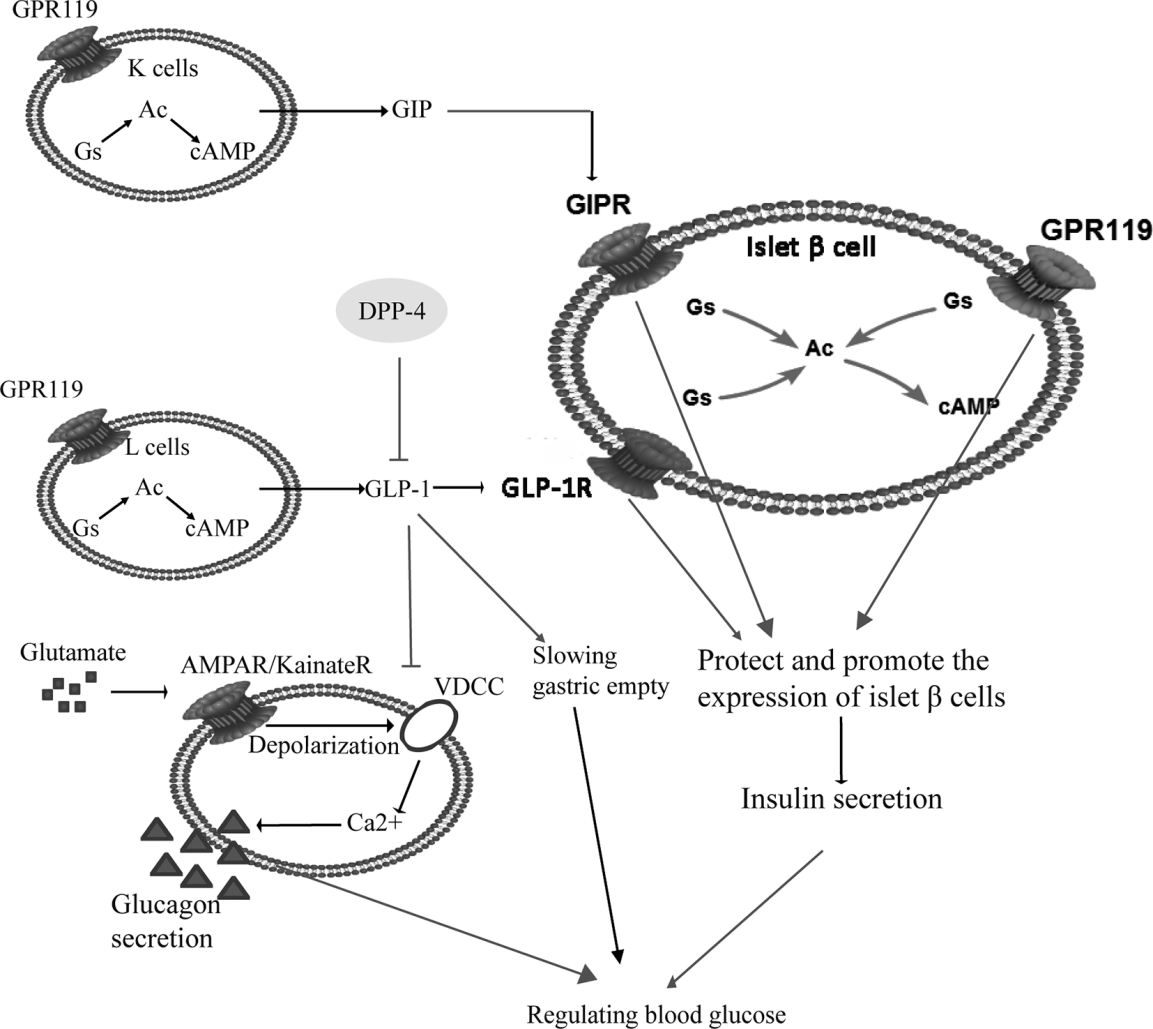
The pathogenesis of type 2 diabetes mellitus (T2DM) and related anti-hyperglycemia targets. Pancreatic β cells secrete insulin, and regulate blood sugar by activation of GLP-1R and GIPR on their surface. In patients with T2DM, after a meal food reaches the gastrointestinal tract and promotes GLP-1 inactivation under the catalysis of DPP-IV. K cells and L cells work together to regulate insulin secretion. GIP and GLP-1 bind with GIPR and GLP-1R respectively, together with GPR119 to protect pancreatic β cells and stimulate the secretion of insulin. Increased GLP-1 inhibit αcells secreting glucagon to lower blood glucose. Meanwhile, GLP-1 reduces brain satiety and slows gastric emptying. Abbreviations: GPR119, G protein-coupling receptor 119; GIP, glucose-dependent insulinotropic polypeptide; GLP-1, glucagon like peptide-1; GIPR, glucose-dependent insulinotropic polypeptide receptor; GLP-1R, glucagon like peptide-1 receptor; Ac, adenylate cyclase; Gs, stimulatory G protein; cAMP, cyclic adenosine monophosphate.

T2DM is also called noninsulin-dependent diabetes mellitus, which is induced by pancreatic β-cell dysfunction and insulin resistance. Initially, there is a compensatory increase in insulin secretion, followed by a decrease in insulin secretion because of the damage of β cells. The initial insulin response to secretagogue is also reduced, which is the first phase of the reaction of chronic hyperglycemia state (Dinneen et al., 1992). For the pathogenesis of T2DM, insulin resistance is the original factor and the damage of pancreatic β cells is the key factor (Gulli et al., 1992; Martin et al., 1992; Defronzo, 2009). Pancreatic β cells belong to islet cells, accounting for 70% of the total number of islet cells, and are mainly located in the central part of the pancreas. They can secrete insulin to regulate blood glucose. A prospective study found that islet function was about 50% of normal at the time of diagnosis, and a reduction in β-cell mass of about 60% was shown at necropsy (Neutzsky-Wulff et al., 2012). Pancreatic β cells play their role by activation of glucagon like peptide-1 receptor (GLP-1R) and glucose-dependent insulinotropic polypeptide receptor (GIPR) on their surface. In T2DM patients, after a meal, food reaches the gastrointestinal tract, stimulates the pancreas and promotes glucagon-like peptide-1 (GLP-1) inactivation under the catalysis action of dipeptide peptidase-IV (DPP-IV). Glucose-dependent insulinotropic polypeptide (GIP) is an incretin synthesized and secreted by the K cells in the duodenum and jejunum. It is mainly stimulated by diet, especially fat. Excessive secretion of GIP leads to the deposition of lipids in peripheral tissues (liver, muscle, etc.) and islet β cells, which causes impaired insulin resistance and secretory function, while inhibition of GIP secretion can significantly improve T2DM associated with obesity. GLP-1 is an incretin synthesized and secreted by distal ileal L cells. Glucose and fat in food are the most important nutrients to stimulate GLP-1 release. GLP-1 can promote glycogen synthesis and insulin expression, delay gastric empty and glucagon secretion, while also promote proliferation of pancreatic β cells (Urbano et al., 2016). Pancreatic β cells also express G protein-coupling receptor 119 (GPR119), which can protect and promote pancreatic β cells to stimulat the secretion of insulin, together with GIPR and GLP-1R. GLP-1R binds to stimulatory G protein (Gs), activates adenylate cyclase (AC), and further catalyzes the production of cyclic adenosine monophosphate (cAMP) by adenosine triphosphate (ATP). cAMP is the first signal transduction molecule that GLP-1 stimulates β cells to produce insulin. Increasing cAMP can increase the insulin stimulation of β cells (Dov et al., 2008). GLP-1 also reduces brain satiety and inhibits gastric empty. They play a common role in regulating blood glucose, as shown in **Figure 1**.

In some patients with mild diabetes, blood glucose can be successfully controlled by exercise and diet without drugs (American Diabetes Association, 1998; Balducci et al., 2014). However, many patients require additional insulin or oral hypoglycemic agents to achieve euglycemia. The nodes of pathogenesis of T2DM become targets of the invention of antihyperglycemic drugs, such as insulin secretagogues, insulin sensitizer agents, α-glucosidase inhibitors, GLP-1 analogs, and DPP-4 inhibitors. However, many side effects have been reported, such as hypoglycemia and gastrointestinal reactions. Therefore, it is necessary to summarize the categories of existing patents and find new drugs to better guide the clinical treatment of T2DM.

**Figure 2:**
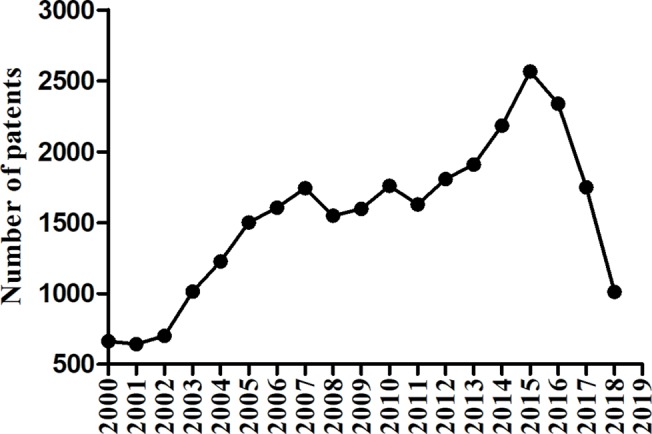
Trend analysis on the population of patents with diabetes mellitus in China.

The number of patents on drugs for diabetes mellitus has been increasing in recent years, even with some decline in the latter years, as shown in **Figure 2**. And many pharmaceutical companies and organizations are devoting themselves to finding or inventing new products. The aim at this review is to document the current drug patents in China of antihyperglycemic, and guide the invention of new drugs and their clinical use.

## Materials and Methods

### Data Retrieval Method

All data were obtained from the website of State Intellectual Property Office of the People’s Republic of China: http://www.sipo.gov.cn. In our searches, we used the keywords “diabetes mellitus,” “diabetes,” “hyperglycemia” “type 2 diabetes mellitus,” and “T2DM.” These keywords can be used either independently or as a phrase by adding “or.” After careful reading, screening, and deleting invalid patents, we have obtained 2,500 patents on antihyperglycemic therapies for T2DM.

### Patent Review

**Figure 3:**
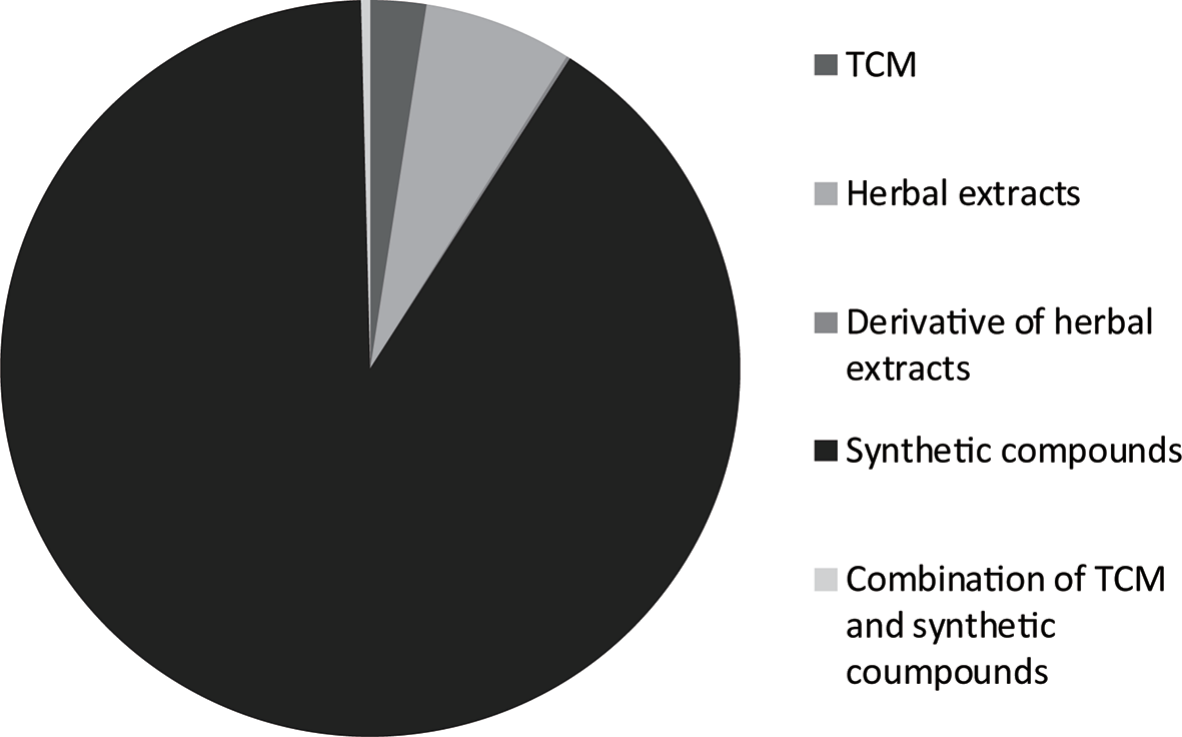
Distribution and composition of traditional Chinese medicine (TCM), herbal extracts, derivatives of herbal extracts, synthetic compounds and combinations of synthetic compounds and TCM patents for anti-T2DM therapies issued in China.

A total of 2,500 patents for anti-T2DM remedies were found. The 2,500 patents consisted of 4 patents for derivatives of herbal extracts (0.2%), 162 patents for herbal extracts (6.5%), 61 compositions for traditional Chinese medicine (TCM) (2.4%), 2,263 patents for synthetic compounds (90.5%), and 10 (0.4%) patents of a combination of synthetic compounds and TCM. The distribution of these patents is shown in **Figure 3**. Compared to TCM, antidiabetic synthetic compounds appeared more often in prescriptions because of their clear pharmacological action and adverse reactions. Currently, patents on antihyperglycemic drugs for T2DM include insulin secretagogues, insulin sensitizer agents, α-glucosidase inhibitors, GLP-1 analogs, DPP-4 inhibitors, cannabinoid receptor type 1 antagonists, and endothelin receptor antagonists (Srinivasan et al., 2008). There are also some new drug targets, such as GPR119 agonists, and G protein-coupled receptor 40 (GPR40) agonists. In the following part of this article, we will describe these categories in details.

#### Insulin Secretagogues

##### Thiazolidinediones

Thiazolidinediones (TZDs) are identified as insulin sensitizers that directly protect islet β cells and promote insulin secretion (Lebovitz and Banerji, 2001; Leiter, 2005; Campbell and Mariz, 2007). Furthermore, TZDs increase the sensitivity of liver, adipose, and muscle tissue to insulin. They also promote hepatic glucose production and inhibit peripheral glucose utilization (Hara et al., 2006; Riera-Guardia and Rothenbacher, 2008).

As a representative TZD, pioglitazone is a “wonder drug” with several functions. In the Actos Now for prevention of diabetes (ACT NOW) study subjects, the results in the pioglitazone group showed that pioglitazone inhibited the progression from IGT to T2DM by 72% (p < 0.001) (Podar et al., 2001; Wentworth et al., 2014). A recent study suggested the combination therapy of pioglitazone and DPP-4 inhibitor provided better glycemic control than pioglitazone monotherapy, and reflected on HbA1c and FPG levels (Wang et al., 2018). As another classical TZD, rosiglitazone has shown its pharmacological effects in preclinical and clinical experiments. Rosiglitazone can improve the insulin resistance, control blood glucose and prevent rebound (Finegood et al., 1999). However, rosiglitazone also causes some adverse effects, such as edema, weight increase and serious osteoporosis (Malinowski and Bolesta, 2000). Therefore, it is necessary to design safe and effective TZDs. A protected patent describes a series of TZD N-substituted derivatives that were synthesized to improve the activity of the TZD ring, reduce side effects and extend its applications (Hu Xiangnan et al., 2012). The invention utilizes the active hydrogen of the nitrogen atom on the rosiglitazone TZD ring without affecting the pharmacological TZD ring, and is chemically modified to link a series of small molecule substituents. The N-substituting derivatives were designed to improve their properties, reduce toxic side effects, and increase their range of application (Hu Xiangnan et al., 2012).

##### Sulfonylureas

Sulfonylureas (SUs) are the earliest and the most frequently used hypoglycemic agents in clinic. They are especially suited for patients who cannot achieve ideal blood glucose even with exercise and diet. SUs act on the SU receptor 1 (Sur1), which is expressed on the surface of pancreatic β cells. When Sur1 is combined with a SU, Ca^2+^ channels are opened and Ca^2+^ flows into intracellular regions to stimulate insulin secretion. This category has the same structure (R_1_-SO4NHCONH-R_2_). Because of its serious side effects, drugs of the first generation like toluene SU were withdrawn (Harrower, 2000). The latter glibenclamide and glimepiride are still on the markets. Glimepiride is a long-acting hypoglycemic agent to stimulate insulin secretion and improve the sensitivity of insulin in the peripheral tissues, with a higher hypoglycemic effect than that of glipizide. Glimepiride can be used alone to control the blood glucoses, especially in patients who cannot control the blood glucose only with diet and exercise. It can also cooperate with other hyperglycemic agents or insulin, with the advantages of high efficiency, long-acting safety, low dose, low toxicity, and fewer adverse effects. However, it is limited by the poor stability low dissolution and bioavailability when glimepiride acts as oral and solid preparation. Consequently, it is necessary to provide a glimepiride solution delivery system with high bioavailability, fast onset, and better stability to obtain better hypoglycemic effect. A protected patent for sublingual administration may solve this problem. Compare with the solution administration system, the patented formulation is administered by spraying to achieve quick effect, high bioavailability and avoid adverse effects on gastrointestinal and liver. It works for postprandial blood glucose control, especially in T2DM patients whose blood glucose cannot be controlled by diet and exercise alone. The manufacturing process is simple, feasible, and suitable for industrial production (Gao Yongliang et al., 2008).

##### Biguanides

Biguanides mainly act on islet tissue to inhibit intestinal epithelial cells from absorbing glucose. It increases the sensitivity of peripheral tissues to insulin, and increase noninsulin-dependent liver glycogen regeneration. Biguanides stimulate the anaerobic glycolysis of glucose, inhibit hepatic gluconeogenesis, and decrease plasma glucagon levels (Miller et al., 2013).

In Chinese, Li et al. (2002) found that metformin can reduce fasting blood glucose and blood glucose 1h after an oral glucose tolerance test (OGTT), and delay the progression to T2DM. Metformin hydrochloride plays a significant role among the guanidine oral hypoglycemic agents in the drug treatment of T2DM. The hypoglycemic effect of metformin is obvious, probably because that metformin hydrochloride increases the uptake and utilization of glucose in peripheral tissue, enhances tissue insulin sensitivity, inhibits gluconeogenesis and decomposition, reduces the high glycogen output, and delays the absorption of glucose in the intestine. It can reduce glycosylated hemoglobin and the free fatty acid concentration without stimulating insulin secretion from pancreatic β cells and causing premature failure of pancreatic β cells (Yang et al., 2017). When the blood glucose level is regular, there will be no hypoglycemic effect and the weight gain. However, there are also other hypotheses suggesting that metformin hydrochloride may improve the sensitivity of insulin (Giannarelli et al., 2003).

By a pathway depend on peroxisome proliferator-activated receptor-α (Maida et al., 2011), biguanides regulate the transcription of insulin-responsive genes to improve the insulin tolerance and have a hypoglycemic effect. There is one patent combines metformin hydrochloride and pioglitazone hydrochloride in a sustained-release pellets preparation. The pellet capsule formulation is a polydispersion system, with the advantages of simple production processes, large drug loading, great liquidity (Ali et al., 2009). When pioglitazone hydrochloride is combined with metformin hydrochloride, it enhances the sensitivity of tissue to insulin, and improves anaerobic glycolysis in peripheral tissues. The two hypoglycemic agents are complementary regarding onset and duration of action, mutual overlap effects, offsetting side effects. The combination of metformin and pioglitazone is an ideal treatment for early phase of T2DM to prevent insulin resistance (Perez et al., 2009).

#### α-Glucosidase Inhibitors

α-Glucosidases are catalyticase to catalyze the hydrolysis of α-glucosidase groups from the non-reducing ends of α-glucoside substrate. They are widely distributed among organisms and participate in food digestion, glycoprotein biosynthesis. α-Glucosidase inhibitors are oral hypoglycemic agents that delay the absorption of intestinal carbohydrates and achieve effective treatment of T2DM. α-glucosidase inhibitors can competitively inhibit the various α-glucosidases to inhibit glucose absorption in the small intestine. Studies have shown that α-glucosidase inhibitors can improve postprandial hyperglycemia and alleviate hyperinsulinemia while increasing glucose tolerance (van de Laar, 2008).

Tan et al. extracted one kind of α-glucosidase inhibitor from akebia plants, the new 23, 29-drop oleanolic acid compounds. Akebia plants are a rich source of these compounds, and can be used to extract the medicine when the fruit is harvested. Its activity in inhibiting α-glucosidase is five times greater than that of acarbose. Acarbose is currently a first-line drug for T2DM. Therefore, the new 23, 29-drop oleanolic acid compounds are highly likely to become marketed drugs (Tan Jianwen et al., 2013).

#### GLP-1 Agonist

GLP-1 is secreted from gastrointestinal L cells (Theodorakis et al., 2006) and appears in the plasma within several minutes after ingestion of food (Herrmann et al., 1995; Orskov et al., 1996). GLP-1 regulates plasma glucose in the postprandial period by stimulating insulin secretion, inhibiting glucagon secretion and slowing gastric emptying (Komatsu et al., 1989; Flint et al., 2001; Holst and Gromada, 2004; Drucker, 2006; Hare et al., 2010). The effect of GLP-1 is initially mediated by its effect delaying gastric emptying, which leads to delayed entry of nutrients into the circulation (Chen and Yang, 2014). The effect of incretin drops off in T2DM patients, which contributes to postprandial hyperglycemia (Holst et al., 2011). GLP-1 agonists help stabilize glycemic control.

Exenatide is a member of the GLP-1 family. It can bind to and activate GLP-1R. GLP-1R is involved in the synthesis of glucose-dependent insulin by intracellular signaling mechanisms and stimulates β cells secreting insulin. Exenatide can promote insulin release from β cells and improve blood glucose control in patients with T2DM by controlling fasting and postprandial blood glucose levels (Grossman, 2014). However, the half-life of GLP-1 in the serum is only 3 to 5 min, that limits clinical applications. A patent invented by Li et al. in Tianjin Sanli Patent Co., Ltd showed a GLP-1 analogue, of which some amino acids were replaced by cysteine. It has a half-life of 72 to 96 h, much longer than that of GLP-1 itself. This finding created the foundation for the widespread clinical use of GLP-1 analogues (Li Ying and Yuan, 2014).

#### DPP-4 Inhibitors

GIP and GLP-1 both stimulate insulin secretion, and can be hydrolyzed by DPP-4. To date, seven DPP-4 inhibitors have been sequentially listed worldwide: sitagliptin, vildagliptin, saxagliptin, alogliptin, linagliptin, gemigliptin and teneligliptin. In 2009, the European Medicines Evaluation Administration (EMEA) approved the use of saxagliptin in combination with metformin, TZDs, or SUs to treat T2DM (Dhillon, 2015).

Saxagliptin is a powerful and selective DPP-4 inhibitor which can specifically prolongs the inhibitory effect of DPP-4, prolongs endogenous GLP-1 and GIP duration, and reduces blood glucose (Augeri et al., 2005; Zhao et al., 2005). The most frequent adverse reactions reported during the phase III trial of pravastatin were headache, upper respiratory tract infection, and urinary tract infection. In combination therapies, the incidence of adverse reactions to saxagliptin and placebo was similar (Defronzo et al., 2009; Hollander et al., 2009). The National Institute for Health and Clinical Excellence (NICE) recommended that DPP-4 inhibitors be considered the first-line treatment for patients with T2DM who are unable to tolerate metformin or SUs when the patient has significant risk of hypoglycemia (Adler et al., 2009).

A new compound invented by Zhang Xiaoqing et al. in 2012 (Zhang Xiaoqing et al., 2012) inhibits the activity of DPP-4. It is an amidation reaction of existing DPP-4 inhibitors and their derivatives to prepare novel polymer DPP-4 inhibitors. Compared with other DPP-4 inhibitors, it has higher inhibitory activity and provide a new choice for the treatment of T2DM.

#### Cannabinoid-1 Receptor Inhibitors

The cannabinoid-1 (CB1) receptors distribute among the brain and other tissues. A study showed that activation of the CB1 receptor increased feeding behavior. Therefore, CB1 receptor antagonists have been developed as potential agents to treat obesity and other metabolic diseases, such as T2DM. Rimonabant, a CB1 receptor antagonist, has effects on controlling obesity and the metabolic syndrome (Carai et al., 2005; Van Gaal et al., 2005). A study in North America showed that 20 mg rimonabant everyday could lose weight, decrease waist circumference and triglycerides, increased high-density lipoprotein (HDL) cholesterol, and hindered the progress of metabolic syndrome (Pi-Sunyer, 2006). Currently, there is no patent for a CB1 receptor inhibitor in the protected period, which suggests its limited clinical value and uncertain future.

#### G Protein-Coupled Receptor 40 Agonists

GPR40 is reported to be expressed in the brain, with unclear functions and mechanisms at present. GPR40 is activated by endogenous ligands such as linoleic acid, decanoic acid, and docosahexaenoic acid (DHA) (Katayama et al., 1994; Nakamoto et al., 2011). Until now, many synthetic patents of GPR40 agonists have been reported and the optimal compound was TAK-875 from Takeda Pharmaceutical Company (Negoro et al., 2010). However, due to its signs of hepatotoxicity in patients, phase III clinical trials involving TAK-875 was finally discontinued (Mohammad, 2016). In the Langerhans islets isolated from GPR40 knockout mice, the glucose-responsive insulin secretion-promoting effect of fatty acids was reduced compared to normal mice (Zhang Yingjun et al., 2014). A new GPR40 agonist was developed by Zhang *et al*. The invention provides a composition which has good stability and rapid dissolution. It contains the active ingredient 2-((S)-6-((2’,6’-dimethyl-4’-((2’,6’-dimethyl-4’-(((R)-tetrahydrofuran-3-yl)oxy)-[1,1’-biphenyl]-3-yl)methoxy)-2,3-dihydrobenzofuran-3-yl)acetic acid or medicinal salts, and polyvinylpyrrolidone. When the amount of polyvinylpyrrolidone in the composition is from 0.5 to 20% by weight, it has good compressibility and fluidity. It has the advantages of good solubility and stability (Zhang Daimei and Tingting, 2017).

#### GPR119 Agonists

GPR119 is a G protein-coupled receptor expressed on β cells, L cells and K cells predominantly in the pancreas and gastrointestinal tract. GPR119 has revealed two classes of possible endogenous ligands, phospholipids and fatty acid amides, of which, fatty acid amides have attracted attention due to its known effects of reducing food intake and losing weight. GPR119 agonists can increase intracellular cAMP levels. GPR119 can also stimulate insulin-releasing by enteroendocrine cells. The effects of GPR119 agonists in animal models of diabetes and obesity were reviewed by Overton et al. (2008), and the potential value of these compounds in T2DM therapies are discussed (Overton et al., 2008).

#### Other Chinese Medicines

Treatment of diabetes mellitus has a long history by TCM. The first patent for a TCM to treat diabetes mellitus was issued in 1999. The preparation consisted radix bupleuri, mulberry leaf, mulberry, radix astragali, radix puerariae, fructus lycii, and other ingredients (Gang, 1998). Subsequently, Liu Xin and Kun (2009) produced a powder containing ginseng, rhizoma anemarrhenae, gypsum, bitter gourd, cocoon, astragalus, etc. Tan Xiaozhong et al. (2015) prepared a powder containing ginseng, poria, atractylodes, radix rehmanniae, radix astragali, yam, etc. These herbs are in accordance with the TCM principle of gout “nourishing yin, replenishing qi, tonifying kidney, promoting blood circulation and removing blood stasis.”

**Figure 4:**
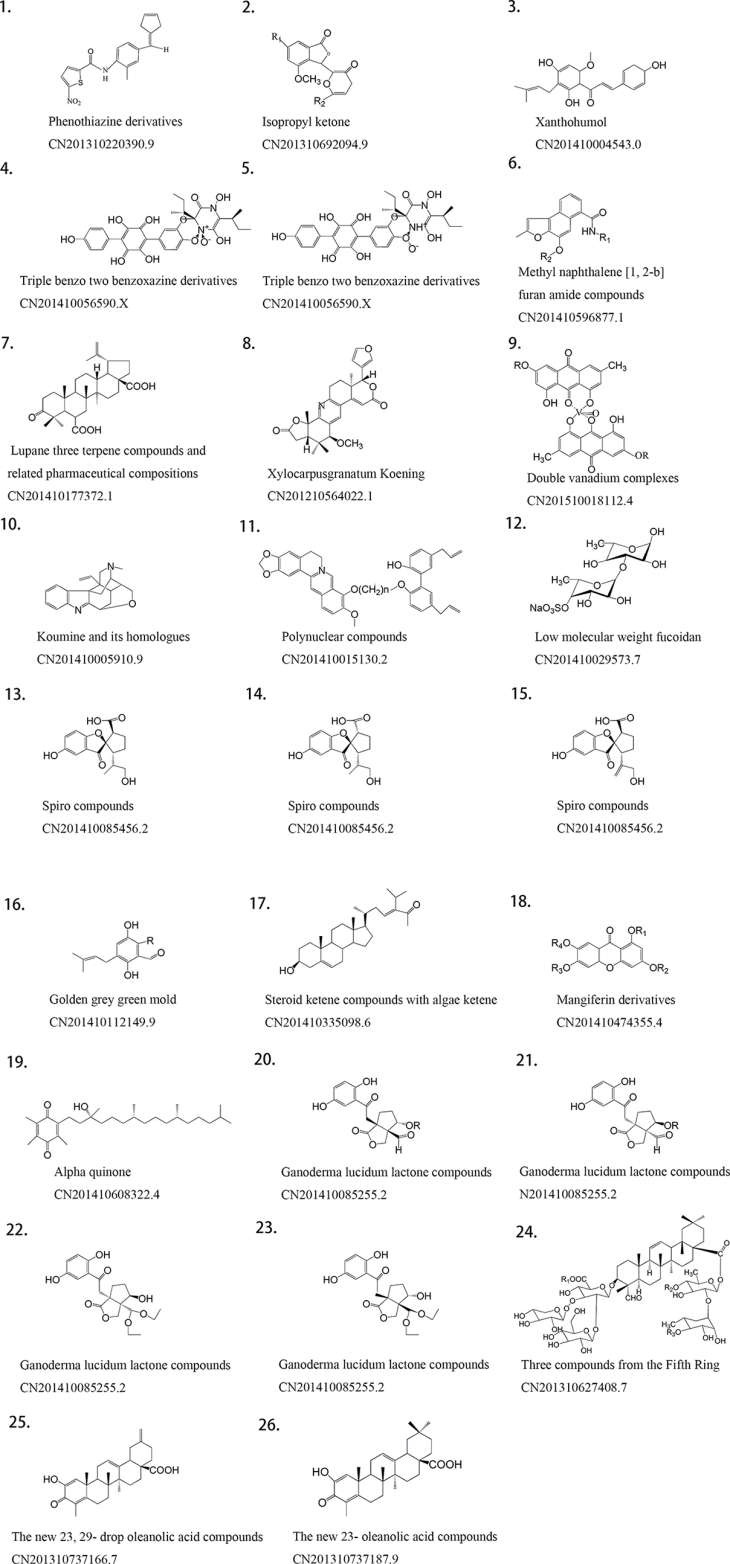
Structure of active phytochemical ingredients of herbal extracts in anti-diabetic properties in Chinese patents.

Among 61 TCM patents, 20 active ingredients are the most frequently mentioned (**Table 1**, **Figure 4**). The unclear mechanisms limit their widespread application, and a discussion of those issues is beyond the scope of this review.

**Table 1:** Twenty patents on phytochemical ingredients of herbal extracts in anti-diabetic properties, and their natural sources, and their protected content.

Natural material	Effective component	Patent protection content	Patent
Eclipta	Phenothiazine derivatives	Compound extraction process, new use	CN201310220390.9 (Chen Wansheng et al., 2013)
Mangrove	Isopropyl ketone	Compound, preparation, process, new use	CN201310692094.9 (She Zhigang et al., 2013)
Hops	Xanthohumol	Compound, new use	CN201410004543.0 (Liu Ming and Ge, 2014)
*Sarcodon leucopus*	Triple benzo two benzoxazine derivatives	Compound, new use	CN201410056590.X (Liu Hongwei et al., 2014)
*Stellera chamaejasme*	Methyl naphthalene [1, 2-b] furan amide compounds	Compound, new use	CN201410596877.1 (Chen Ye et al., 2014)
Coral	Lupane three terpene compounds and pharmaceutical compositions thereof	Compound, preparation, process, new use	CN201410177372.1 (Xu Gang et al., 2014)
*Xylocarpus granatum* Koening	Meliaceae alkali corydaline	Compound, extract, extraction process, new use	CN201210564022.1 (Li Jia et al., 2012)
*Polygonum cuspidatum*	Double vanadium complexes	Compound, new use	CN201510018112.4 (Ning Guilin et al., 2015)
Akebia	The new 23- oleanolic acid compounds	Compound, extraction process	CN201310737187.9 (Tan Jianwen et al., 2013)
Gelsemine	Koumine and its homologues	Compound, new use	CN201410005910.9 (Yu Changxi et al., 2014)
Gelsemine	Polynuclear compounds	Compound, extraction process	CN201410015130.2 (Li Weimin et al., 2014)
Fucus	Low molecular weight fucoidan	Extraction process, new use	CN201410029573.7 (Ji Aiguo et al., 2014)
*Ganoderma lucidum*	Spiro compounds	Compound, extraction process, new use	CN201410085456.2 (Cheng Yongxian et al., 2014)
Fungus	Golden grey green mold	Compound, new use	CN201410112149.9 (Zheng Zhihui et al., 2014)
Caulerpa racemosa	Steroid ketene compounds with algae ketene	Compound, extraction process, new use	CN201410335098.6 (Mao Shuichun et al., 2014)
Mango	Mangiferin derivatives	Compound, new use	CN201410474355.4 (Liu Yidan et al., 2014)
*Caulerpa racemosa*	Alpha quinone	Extraction process	CN201410608322.4 (Mao Shuichun et al., 2014)
*Ganoderma lucidum*	Ganoderma lucidum lactone compounds	Compound, extraction process, new use	CN201410085255.2 (Cheng Yongxian et al., 2014)
Tile grass	Three compounds from the Fifth Ring	Compound, new use	CN201310627408.7 (Wang Xueyong, 2013)
Akebia	The new 23 29- drop oleanolic acid compounds	Compound, extraction process	CN201310737166.7 (Tan Jianwen et al., 2013)

## Conclusions

Our search found 2,500 published patents, including 4 patents of derivatives of herbal extracts (0.2%), 162 patents for herbal extracts (6.5%), 61 compositions for traditional Chinese medicine (TCM) (2.4%), 2,263 patents of synthetic compounds (90.5%), and 10 (0.4%) patents of a combination of synthetic compounds and TCM. There are fewer patents for derivatives of herbal extracts and herbal extracts themselves than the others, especially synthetic compounds which target islet β cells, α-glucosidase, and GLP-1. We expect more compounds and more effective preparations could be developed to improve therapeutic benefits for T2DM patients.

### Expert Opinion

In view of the global spread of T2DM and the actual low rate of blood glucose compliance, clinicians should take more actions to address this problem. These include lower hypoglycemic targets [glycosylated hemoglobin (HbA1c) <6.5%], earlier drug therapy, more frequent detection, and more drug options such as GLP-1 analogs and DPP-4 inhibitors.

Clinicians should determine the utility of different drugs by analyzing drug safety, hypoglycemic strength, hypoglycemic risk, expected patient compliance, and total treatment costs (not just drug costs). The treatment choices depend primarily on the level of HbA1c at the patient’s baseline and previous treatments. For the treatment of patients with HbA1c ≤7.5%, monotherapy can reduce HbA1c to ≤6.5%. The antihyperglycemic drugs include metformin, TZDs, DPP-4 inhibitors, and α-glucosidase inhibitors.

Insulin sensitivity and β-cell function can be improved by weight loss to prevent prediabetes into T2DM. However, 40–50% patients with IGT still progress to T2DM, even after successful long-term weight loss. Chemotherapy provides an alternative strategy to improve insulin sensitivity and preserve β-cell function. Combination therapy is the best treatment for T2DM. Combination therapy are complementary to reduce the dosage of each drug and avoid side effects, which can be to better adapt to patients with changing conditions. Classical combination therapies include SUs plus biguanides or glucosidase inhibitors, biguanides plus glucosidase inhibitors or insulin sensitizers, insulin treatment plus biguanides or glucosidase inhibitors and others. The general principle of combination therapy is that two kinds of drugs with different mechanism of hypoglycemic action are selected jointly, and the combination of three types of hypoglycemic drugs is not recommended. The final aim of clinical pharmacy is to achieve an individual medication regimen for each patient. We expect the increasing number of patent submissions for antihyperglycemic therapies soon. Our study could help better catch the opportunities in research on antihyperglycemic drugs in China and promote the development of appropriate antihyperglycemic therapies.

## Author Contributions

JFW and HY designed the article. JFW revised the article. WZ collected the data, analyzed the data, and wrote this review. The other authors collected data and revised the article.

## Funding

This project was sponsored by the grants from the National Natural Science Foundation of China (81571568 and 81871265); CAMS Innovation Fund for Medical Sciences (CIFMS:2016-I2M-1003); and Innovation wTeam of Jiangsu Provincial Commission of Health and Family Planning (CXTDA2017049).

## Conflict of Interest Statement

The authors declare that the research was conducted in the absence of any commercial or financial relationships that could be construed as a potential conflict of interest.
